# Broader HIV-1 neutralizing antibody responses induced by envelope glycoprotein mutants based on the EIAV attenuated vaccine

**DOI:** 10.1186/1742-4690-7-71

**Published:** 2010-09-01

**Authors:** Lianxing Liu, Yanmin Wan, Lan Wu, Jianping Sun, Huiguang Li, Haishan Li, Liying Ma, Yiming Shao

**Affiliations:** 1 State Key Laboratory for Infectious Diseases Prevention and Control, National Center for AIDS/STD Control and Prevention, Chinese Center for Disease Control and Prevention, 155 Changbai Road, Changping District, Beijing 102206, China; 2 State Key Laboratory of Virology, Wuhan Institute of Virology, Chinese Academy of Sciences, No. 44 Xiaohongshan Road, Wu Chang District, Wuhan 430071, China; 3 Department of Medicine, University of California, San Francisco, San Francisco, CA 94143, USA; 4 Science Research Department, Shanghai Public Health Clinical Center, Public Health Clinical Center affiliated to Fudan University, Shanghai, MD 201508, China

## Abstract

**Background:**

In order to induce a potent and cross-reactive neutralizing antibody (nAb), an effective envelope immunogen is crucial for many viral vaccines, including the vaccine for the human immunodeficiency virus (HIV). The Chinese equine infectious anemia virus (EIAV) attenuated vaccine has controlled the epidemic of this virus after its vaccination in over 70 million equine animals during the last 3 decades in China. Data from our past studies demonstrate that the Env protein of this vaccine plays a pivotal role in protecting horses from both homologous and heterogeneous EIAV challenges. Therefore, the amino acid sequence information from the Chinese EIAV attenuated vaccine, in comparison with the parental wild-type EIAV strains, was applied to modify the corresponding region of the envelope glycoprotein of HIV-1 CN54. The direction of the mutations was made towards the amino acids conserved in the two EIAV vaccine strains, distinguishing them from the two wild-type strains. The purpose of the modification was to enhance the immunogenicity of the HIV Env.

**Results:**

The induced nAb by the modified HIV Env neutralized HIV-1 B and B'/C viruses at the highest titer of 1:270. Further studies showed that a single amino acid change in the C1 region accounts for the substantial enhancement in induction of anti-HIV-1 neutralizing antibodies.

**Conclusions:**

This study shows that an HIV envelope modified by the information of another lentivirus vaccine induces effective broadly neutralizing antibodies. A single amino acid mutation was found to increase the immunogenicity of the HIV Env.

## Background

Both EIAV and HIV are members of the *Lentivirus *genus of the Retroviridae family [1,2]. Although the clinical manifestations of infections by EIAV and HIV are different, the underlying mechanisms of persistence and pathogenesis are very similar [3,4]. These similarities are based on the common genetic organization, the molecular mechanism of viral replication, and the conformational structures of the viral structural proteins [5-9]. Most chronically infected horses survive the subclinical carrier phase after recurring cycles of fever, anemia, weight loss, and thrombocytopenia [10,11]. Therefore, EIAV has been used as a model to study HIV-1 persistence, pathogenesis, and immune responses [12-17].

Despite many years of ongoing research, an effective HIV vaccine has not yet been developed. The first successful lentivirus vaccine was an EIAV vaccine, which was made 30 years ago [18,19]. Therefore, the EIAV vaccine can serve as a good model to identify the mechanisms of immune responses against lentiviruses and shed light on how to design an effective HIV vaccine. Studies on the animal models of EIAV, FIV, and SIV showed that attenuated vaccines can be highly effective against infection by wild-type strains [18-22]. The Chinese EIAV donkey-leukocyte attenuated vaccine (DLV) was developed through long-term tissue culture attenuation (123 passages) from a highly pathogenic EIAV strain D510. The latter was obtained from *in vivo *passages (17 and 117 passages in horses and donkeys respectively) of a field EIAV isolates, LN40 strain. The DLV vaccines have turned out to be effective, with about 80% of vaccinated horses resisting challenge by homogeneous and heterogeneous virulent EIAV strains [18,19].

The envelope protein of EIAV plays a pivotal role in the receptor binding on target cells, the subsequent entry into the cell, and the induction of humoral immune responses [23-25]. Previous work with EIAV, FIV as well as SIV has shown that there is a progressive maturation of Env-specific antibody responses to various attenuated lentiviral vaccines [15,26-28]. The mature immune responses including high titer and high avidity can be enhanced by a modified Env, leading to protective vaccine immunity [15,26-29]. Towards this objective, the current studies were conducted. We enhanced the immunogenicity of the HIV Env by making certain envelope mutations associated with the effective EIAV vaccine.

## Results

### Vaccines Construction

**Figure 1:**
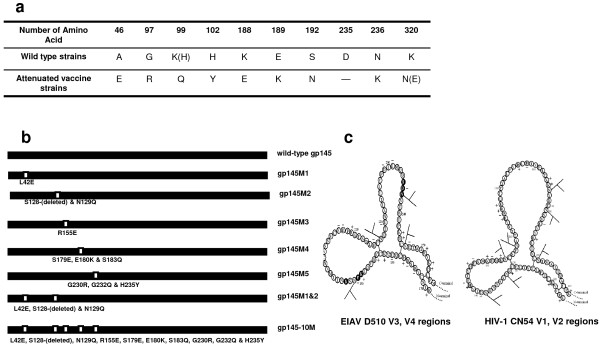
**Consensus mutations and schematic structures are similar between EIAV and HIV-1**. a) Sequence analysis show 10 consensus amino acid mutational sites that have been identified between two Chinese vaccine-derived wild-type EIAV strains and two vaccine virus strains in the EIAV Env region ("--" means that this amino acid was deleted). b) Schematic illustration of gp145 mutants. The figure after the M represents the region of mutations made in the CN54 gp145. c) Schematic figure of the EIAV D510 V3, V4 regions and the HIV-1 CN54 V1, V2 regions. The left figure shows the EIAV V3, V4 regions; the right figure shows the HIV-1 V1, V2 regions. N-Glycosylation sites are shown as branched lines.

From the sequence analysis of two Chinese vaccine-derived wild-type EIAV strains (LN40 and D510) and two vaccine virus strains (DLV and FDDV), 10 consensus amino acid mutations were identified in the EIAV Env region [2] (Figure 1a). We modified the HIV-1 gp145 DNA vaccine and recombinant vaccinia vaccine by introducing all of the EIAV amino acid mutations (Table 1 and Figure 1b). They were based on the structural information of the attenuated EIAV vaccine [5,6] (Figure 1c). We used the gp145 derived from CN54 [Genbank: AX149771], which belongs to the most prevalent CRF BC_07 in China [30], as the template. Details on these constructions are provided in the Methods.

**Table 1:** List of the primers used in PCR for modification

Name	Primer sequence (5'-3')
CN54145F	GCTCTAGAGATATCGACACCATGGACAGGGCCAAGCTGCTGCTG
CN54145R	GTGAACAGGGTGAGGCAGGGCTACTGAGGATCCGTCGACCG
145M1u	ACCACC**GAG**TTCTGCGCCAGCGACG
145M1d	CGCAGAA**CTC**GGTGGTGGTGGCGCCCTTCCACACGG
145M2u	AAC**CAG**GACACCTACCACGAGACC
145M2d	CTCCTCGTGGTAGGTGTCCTGGTTG**CTG**CTCACGTTCCT
145M3u	ACCGTGGTG**GAG**GACAGGAAGCAGAC
145M3d	TTCCTGTC**CTC**CACCACGGTGGTGGCGTTG
145M4u	CTAC**GAGAAG**AACAGC**CAG**GAGTACTACAGGCTGATC
145M4d	C**CTG**GCTGTT**CTTCTC**GTAGTTCTTCTTGGT
145M5u	ATCTTCAAC**CGC**ACC**CAG**CCCTGC**TAC**AACGTGAGCACCG
145M5d	GTT**GTA**GCAGGG**CTG**GGT**GCG**GTTGAAGATCTTGTC

All mutations in primers are marked with bold text.

### Gp145-10 M enhanced the humoral immune responses Env-specific binding antibody responses

**Figure 2:**
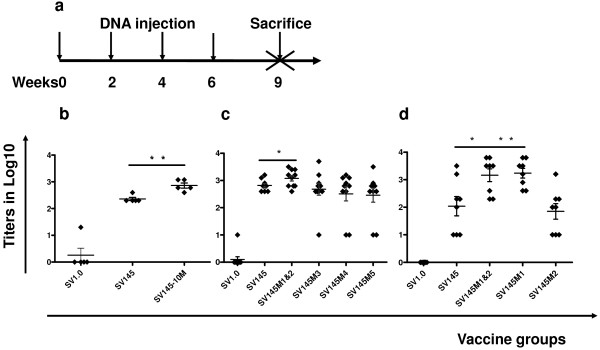
**Specific binding antibody titer**. a) Vaccine inoculation schedule of mice. All groups were inoculated with DNA vaccine at Weeks 0, 2, 4 and 6 and then sacrificed at Week 9 to assess cellular and humoral immune responses. b, c & d) The specific binding antibody titer induced by DNA vaccines. Antibody reactivity was then determined by measuring the optical density (OD) at 492 nm, and endpoint titers were determined by the last dilution whose OD was >two-times than that at the average corresponding dilution of mice sera immunized with SV1.0. The Y value is the log value of the endpoint titers. The significance of differences among the different groups was calculated using a statistical method of one-way analysis of variance (GraphPad prism4.0); * p < 0.05, ** p < 0.01. We obtained data from three experiments using fresh sera.

BALB/c mice were immunized four times with the DNA vaccine SV1.0, SV145, and SV145-10 M at intervals of two weeks and were sacrificed at three weeks after the last inoculation (Figure 2a). The sera of the SV145-10 M group produced binding antibodies at a titer of 1:800. This amount of antibodies was 3.5 times higher than that elicited by SV145 (P = 0.0020). The mock vector (SV1.0) control group only generated a background of antibodies at <1:100 (Figure 2b).

### Neutralizing antibody responses in BALB/c mice

Neutralizing antibodies were determined with HIV-1 primary isolates (B'/C clade isolates XJDC6371, XJDC6431, XJDC0793, CBJB105, CBJB248 and B' clade isolate 020201300). The serum neutralization titer was determined by assessing whether the sera could neutralize 50% of the virus in triplicate. If the value of 1/50% neutralization titer is 6, it means the sera neutralized more than 50% of the virus at the dilution of 1:6. Sera from all gp145 immunized mice failed to neutralize any of the B'/C clade isolates even at the lowest titer of 1:6. Notably, the sera of the gp145-10 M immunized mice neutralized all five of the B'/C clade primary viruses and virus 020201300. Moreover, all mice neutralizing antibody titers from the gp145-10 M group were higher than 1:12 and neutralized B'/C clade viruses XJDC6431, XJDC0793, CBJB105, CBJB248 higher than 1:24 (Table 2).

**Table 2:** Neutralization titer against HIV-1 clinical isolates in BALB/c mice

Vaccine groups	Neutralization titer against HIV-1 isolates					
	XJDC6371	XJDC6431	XJDC0793	CBJB105	CBJB248	020101300
SV145	< 6	< 6	< 6	< 6	< 6	> 12
SV145-10M	> 12	> 24	> 24	> 24	> 24	> 12
SV145M1&2	< 6	> 24	> 24	> 24	> 24	> 24
SV145M1	< 6	> 24	> 24	> 24	> 24	> 24
SV145M2	< 6	> 12	> 12	> 12	> 12	> 12
SV145M3	< 6	< 6	< 6	< 6	> 12	> 12
SV145M4	< 6	< 6	< 6	< 6	> 12	> 12
SV145M5	< 6	< 6	< 6	< 6	< 6	< 6

The neutralization was conducted by using a panel of clinical isolates in PBMCs with 50% inhibitory dose. Gp145-10 M, gp145M1&2, gp145M1 and 145M2 groups could neutralize HIV-1 isolates at the highest titer of 1:24, other than XJDC6371. The single N-glycosylation site deletion in the V1 loop designated as gp145M2 could induce broader neutralizing antibodies against five clinical isolates at a titer of 1:12.

### Neutralizing antibody responses in guinea pigs

**Figure 3:**
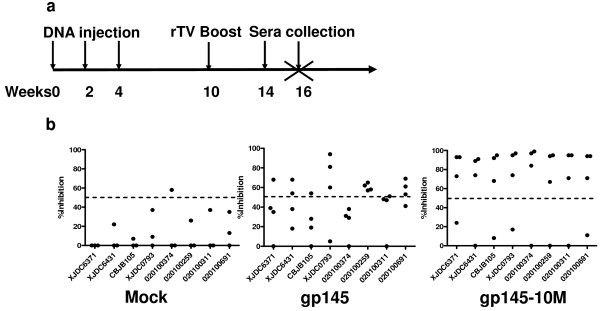
**Inhibition of HIV by sera of immunized guinea pigs at the titer of 1:10**. a) Vaccine inoculation schedule of guinea pigs. All groups were inoculated with DNA vaccine at weeks 0, 2, 4 and then boosted with rTV at week 10. Sera after last immunization were collected at week 14 and 16. b) Comparative inhibition of HIV-1 infection by sera collected at week 16 from mock-, gp145- and gp145-10M-immunized guinea pigs. The neutralizing experiment was conducted by using a panel of clinical HIV-1 isolates from PBMCs in TZM-bl cells. The dotted line in the figure indicates the 50% inhibition rate.

**Figure 4:**
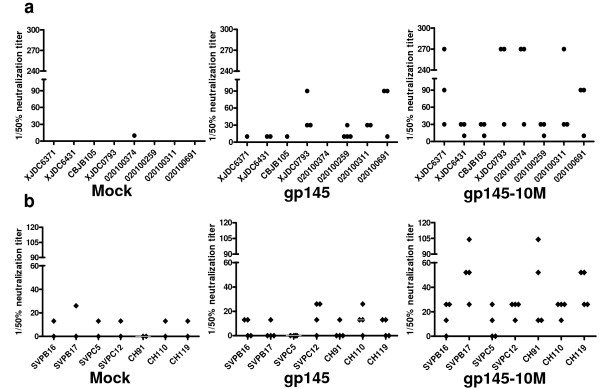
**Neutralizing antibody titers against HIV-1 of immunized guinea pigs' sera**. End-point neutralizing antibody titers of HIV-1 by sera from mock-, gp145- and gp145-10M-immunized guinea pigs. Sera were collected at six weeks after the last immunization for testing. The neutralizing experiment was conducted by using a panel of clinical isolates in PBMC cells (a) and a panel of tier 2-3 peudeovirus in TZM-b1 cells (b). The 50% inhibitory dose (ID50) was defined as the plasma dilution.

Neutralizing antibodies at a titer 1:10 in guinea pigs model were tested at four and six weeks after the last inoculation (Figure 3a). At least three of four sera from gp145-10M-immunized guinea pigs neutralized all of the B'/C clade and B' clade viruses at six weeks (Figure 3b), and similar results were found at four weeks (data not shown). Notably, the neutralization frequency in gp145-10M-immunized animals was 2.5 fold higher than that of gp145-immunized animals at the titer ≥1:10 (Figure 3b). Moreover, at least two of four sera collected from the gp145-10M-immunized guinea pigs showed 80% neutralization at the titer of ≥1:10 (data not shown). In contrast, only one of four sera collected from the gp145-immunized animals had 50% neutralization at the titer of ≥1:10; and only three possible events were found in the gp145-immunized group. Lastly, but most importantly, antibodies induced by gp145-10 M neutralized all HIV-1 isolates and pseudotyped viruses at the highest endpoint titers of 1:270 (Figure 4a,b). The mean neutralization titers from mock-, gp145- and gp145-10M-immunized groups against all viruses were 0, 1:16 and 1:71, respectively (Figure 4a). Two sera of the gp145-10M-immunized guinea pigs neutralized all viruses at titers of 1:98 and 1:158.

### Linear antibody epitope mapping

**Figure 5:**
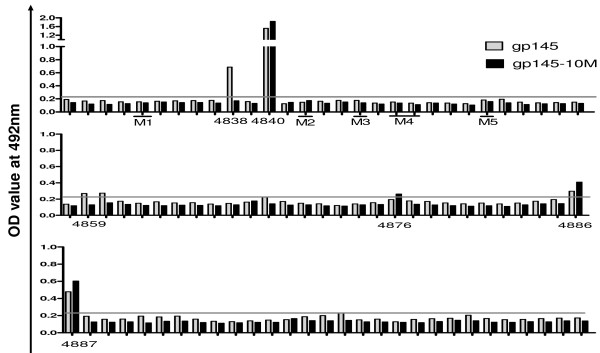
**Linear antibody epitope mapping**. PLL-ELISA was used to detect the responses of the antibody against each linear peptide. The concentration of each peptide of HIVCN54gp145 was 10 μg/ml. In these epitopes, seven were defined as positive (> two-fold background). Epitopes p4838, p4859 and p4860 were only identified in the SV145 group. P4876 was only defined in the SV145-10 M group. The grey line in the figure indicates the threshold value (0.22), which is two times than the average OD values (0.11) from mock controls. Five mutation regions were labeled.

The results of the PLL-ELISA demonstrated that different antibodies to specific linear epitopes were induced among gp145- and gp145-10M-immunized mice (Figure 5). In the C1 region, both gp145 and gp145-10 M induced antibodies to peptide 4840, and the latter enhanced the response. The gp145-10M-immunized animals failed to generate antibodies to peptides 4838, 4859 and 4860, but they induced strong antibody responses to peptide 4876 in the V5 loop and higher antibody titers to peptides 4886 and 4887 in the HR region.

### Env-specific T cell immune responses

**Figure 6:**
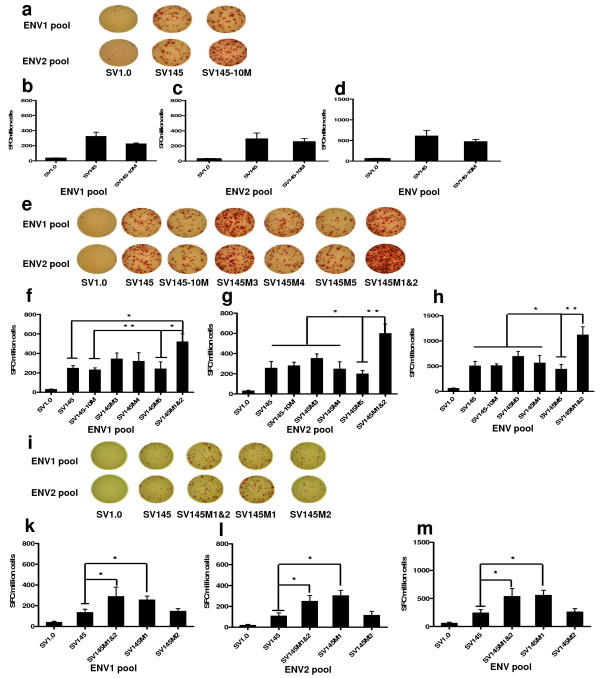
**Specific IFN-gamma secretion detected by ELISPOT**. The T cell immunity was quantified with an IFN-γ-based ELISPOT assay with SHIVchn19 (20-mer) peptides ENV1 (4830-4871) or ENV2 pools (4872-4913). a, e & i) Elispot results in different immunized groups stimulated with stimuli as indicated on the left. b, f & k) Number of SFC in one million splenocytes stimulated by the ENV1 pool. c, g & l) Number of SFC in one million splenocytes stimulated by the ENV2 pool. d, h & m) Env-specific T cell responses against these two peptide pools were compiled together as the total T cell responses for each mouse and were graphed into groups. The mock group generated <50 SFCs/10^6 ^splenocytes. The significance of differences among the different groups was calculated using a statistical method of one-way analysis of variance (GraphPad prism4.0); SFC, spot forming cell; * p < 0.05, ** p < 0.01. The error bars indicate SEM (the standard error of the mean).

HIV-1_cn54 _Env-specific T cell responses were also measured by the IFN-γ-based ELISPOT assay after stimulation of splenocytes with SHIVchn19 peptides from the ENV1 and ENV2 pools. The ENV1 pool is made up of the first 43 peptides (4830-4871), and the others compose the ENV2 pool (4872-4913). The Env peptides of SHIVchn19 are HIV-1 CN54 Env peptides. The data showed no obvious difference between the gp145- and gp145-10M-immunized mice (Figure 6a-d).

### Gp145M1&2 enhanced immune responses Humoral immune responses

Further studies were conducted in mice immunized with gp145M1&2 (composed of mutations of both M1 and M2), gp145M3, gp145M4, and gp145M5 (Figure 1b). Notably, gp145M1&2 (similarly to gp145-10M) induced higher specific binding antibodies than gp145 (p = 0.041). No significant specific binding antibody differences were found in any other group (Figure 2c). Moreover, the sera from the gp145M1&2-immunized animals neutralized almost all of the B'/C isolates and B' viruses at a titer greater than 1:24 (Table 2). The sera of the gp145M3- and gp145M4-immunized groups neutralized HIV-1 isolates CBJB248 and 020101300 with an endpoint titer of 1:12 (Table 2). Overall, gp145M1&2 induced similar potent humoral immunity as gp145-10 M did.

### Env-specific T cell immune responses

The specific cellular responses measured by the IFN-γ ELISPOT assay gave additional results in mice. The gp145M1&2 immunization induced vigorous IFN-γ responses (1116 ± 165 SFC/10^6 ^splenocytes, N = 5), which were significantly higher than those elicited in the gp145 group (627 ± 118 SFC/10^6 ^splenocytes, N = 5) (p = 0.043). A two-fold enhancement of the immune response was achieved by the modification (Figure 6e, h). Although the mutations of gp145M1&2 localized at those epitopes covered by the ENV1 peptide pool, both the ENV1 and the ENV2 peptide pools stimulated higher T-cell immunities in the gp145M1&2-immunized group than in the gp145 and gp145-10M-immunized groups (Figure 6f, g).

### Gp145M1 elicits the best immunity Humoral immune responses

Two new mutants, SV145M1 and SV145M2, were designed to evaluate the enhanced immunogenicity induced by gp145M1&2. (Table 1) The mean specific binding antibody titers were 1:1, 1:666, 1:2900, 1:2800 and 1:279 following immunization with SV1.0, SV145, SV145M1&2, SV145M1 and SV145M2, respectively (Figure 2d). Inoculation with gp145M1&2 and gp145M1 stimulated higher binding antibodies than any other modified gp145. Although the sera from the mice, immunized with gp145M2, neutralized almost all HIV isolates (whether B'/C clade or B clade) at a titer greater than 1:12, the gp145M1-immunized animal sera neutralized viruses at a titer higher than 1:24 (Table 2).

### Env-specific T cell immune responses

The results also showed that Env-specific T cellular immunity was enhanced to a similar level by both the SV145M1&2 and SV145M1 DNA vaccines to the peptides pool of either ENV1 or ENV2 (Figure 6i, k, l &6m). Therefore, gp145M1 appears to be the most important mutation for enhancing the anti-HIV immune response.

## Discussion

The HIV-1 envelope glycoprotein is the primary target for neutralization, and great efforts have been made to enhance the immunogenicity of Env in AIDS vaccine design. Although the primary goal for studies of Env modification is to elicit cross-reactive neutralizing antibodies responses [27,31-38], specific binding antibodies have also been shown to contribute to vaccine-induced protection [28]. Moreover, one study in rhesus monkeys demonstrated that specific T cellular responses elicited by HIV Env contributed to vaccine-induced protection from challenge viruses carrying a heterologous envelope [39]. Similar T cell results were found in the vaccination with the EIAV vaccine EIAVD9 [40]. Another study showed that specific modifications in HIV Env also enhanced the ability to induce broader CD8+ T cell activities [41].

With this information in mind, we performed comparative studies of the structural and functional changes in the Env of the EIAV vaccine and wild-type strains. The critical mutations found in EIAV were introduced into HIV Env based on their conserved secondary structure and glycosylation properties [5,6] (Figure 1). The purpose of the modification was to increase the immunogenicity of HIV Env.

The modified immunogen gp145-10 M (containing all 10 mutations) induced broad and high neutralizing antibody titers both in mice and guinea pigs. All primary HIV-1 isolates could be neutralized by the gp145-10M-immunized sera (Table 2, Figure 3 &4). Additional studies showed similar results following immunization with gp145M1&2 and gp145M1 (Table 2). Both neutralizing antibodies and specific binding antibodies were greatly enhanced in quality as well as in quantity (Figure 2 & Table 2).

**Figure 7:**
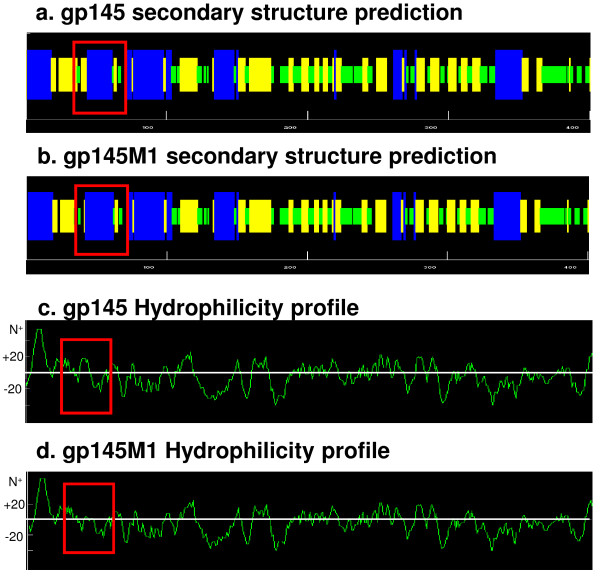
**The secondary structure prediction and hydrophilicity changes of antigens**. AnthePro5.0 software was applied to predict the secondary structure and hydrophilicity changes of gp145 and gp145M1. The red rectangle labels the changed profiles. a & b) The secondary structure predictions of gp145 and gp145M1. α-helix in blue bar; β-sheet in orange bar; β-turn in green bar; others in black bar. c & d) The difference in hydrophilicity between gp145 and gp145M1. The Y-axis shows the value of predicted hydrophilicity.

The PLL-ELISA data showed that antibodies to specific linear epitopes were different between the wild-type gp145- and gp145-10M-immunized groups. However, these different epitopes did not match the mutation sites (Figure 5). We hypothesized that there were conformational changes induced by the modification because the locations of the epitopes were interspersed in the whole envelope. In fact, studies of the secondary structure of these antigens demonstrated one major change in the C1 region caused by the M1 mutation, in which one β-sheet was changed to an α-helix structure compared to gp145 (Figure 7a, b). Furthermore, a 14-amino acid segment within the C1 region that was originally hydrophobic is now amphipathic (Figure 7c, d). These changes may cause the exposure of some conservative epitopes. In this regard, our data suggest that the mutation in the C1 region causes a polarity change of the 14 amino acids and that it induces the exposure of conserved epitopes to stimulate stronger and broader neutralizing antibodies. Other mutations elicit fewer changes in the secondary structure but cause no polarity changes (data not shown).

In addition to the potent neutralizing antibodies and specific binding antibodies observed in the group immunized with gp145M1 and gp145M1&2, high levels of secretion of IFN-γ specific to the ENV1 and ENV2 peptides pools were induced (Figure 6). Because the mutations were modified at the C1 and the V1 regions, the enhancement of specific T cell response to the ENV2 peptides pool was noteworthy. Future studies will focus on the influence of different mutations on the process of antigen presentation [42-45]. Importantly, this study showed that one amino acid change in the C1 region induced potent anti-HIV-1 responses involving specific binding antibodies, neutralizing antibodies, and cellular immunity.

## Methods

### DNA vaccine construction

HIV-1 CN54 isolated in the early prevalence of HIV-1 in China may be considered as an ancestor strain, though the sequence data to establish this concept remains unavailable. The gp145 gene was acquired using PCR amplification from the codon-optimized gp160 gene of CN54. Then, the SV145 plasmid containing gp145 gene was constructed using vector SV1.0 (pDRVI SV1.0). The SV1.0 vector carries a kanamycin resistance gene and a 72-bp element of the SV40 enhancer. Consensus amino acid mutations were identified in the EIAV Env from the sequence analysis of two wild-type EIAV strains, LN40 and D510, and two vaccine virus strains, DLV and FDDV (vaccine developed through 12 passages in fetal donkey dermal cells from DLV). The amino acid modification sites of gp145 were determined by aligning the envelope of EIAV and HIV-1 CN54 with the sequence of the amino acid, the structure of the variable loop and conserved regions, the location of conserved glycosylation sites and the intra-chain disulfide bond sites [1,5,6,46-48] (Figure 1c). Mutants of gp145 were generated by site-directed mutagenesis with two pairs of the primers (Table 1). The resulting vaccines were designated as SV145M1, SV145M2, SV145M3, SV145M4, SV145M5, SV145M1&2 and SV145-10 M, respectively. SV145M1 contains the first mutation L42E; SV145M2 contains mutations S128-(deleted) and N129Q; SV145M3 contains one mutation R155E; SV145M4 contains three mutations S179E, E180K and S183Q; SV145M5 also contains three mutations G230R, G232Q and H235Y. SV145M1&2 was composed of mutations of M1 and M2. SV145-10 M contains all the ten mutations, respectively (Figure 1b). All constructs were confirmed by double-strand gene sequencing.

### Recombinant Tiantan vaccinia vaccine (rTV) construction

Gp145 and gp145-10 M genes were cloned into the pSC65 shuttle plasmid (with a Lac Z gene as a screening marker), which was designed to recombine specifically with the TK gene of the Tiantan vaccinia virus. Subconfluent monolayers of 143B cells were grown in Eagle's media containing 10% fetus bovine serum (FBS) and 1% Penicillin-Streptomycin-L-glutamine. The cells were washed with Eagle's media in the absence of FBS. Five million pfu vaccinia viruses were inoculated 143B cells for one hour at 37°C and 5.0% CO_2_. Thereafter, the vaccinia-infected cells were further transfected with recombinant shuttle plasmids with Lipofectamine 2000 (CAT#11668-019, Invitrogen). After a 48-hour incubation, the transfection medium was removed and all wells were covered with 2% melted low-melting temperature (LMP) agarose mixed with an equal volume of 2× Eagle's media containing 100 μg/ml x-gal. The blue Lac Z-positive colonies were picked up and further purified in 143B cells under the pressure of a selection media (Eagle's media containing 50 μg/ml BrdU). The purified recombinant Tiantan vaccinia viruses were confirmed by PCR amplification of the inserted gp145, gp145-10 M. The generated vaccines were designated as rTV145 and rTV145-10 M. All rTVs were expanded in primary chicken embryo cells.

### Immunization of BALB/c mice

Female BALB/c mice (six weeks old, 18-22 g) were purchased from the Institute of Laboratory Animal Science, Chinese Academy of Medical Sciences & Peking Union Medical College. All animals experiments were reviewed and approved by the Institutional Animal Care and Use Committee (IACUC) at the China CDC animal facility and were performed in accordance with relevant guidelines and regulations. A sample of 100 μg of purified plasmid DNA was suspended in 100 μl of PBS and inoculated intramuscularly into the tibialis anterior four times at intervals of two weeks. The mice were sacrificed at three weeks after the last inoculation. Splenocytes were freshly collected for Elispot assays, and sera were collected and stored at 4°C and -80°C for future quantification of antibodies.

### Immunization of guinea pigs

Female Huntley guinea pigs (six weeks old) were purchased from the Center of Laboratory Animal Science, National Institute for the Control of Pharmaceutical and Biological Products. All animal experiments were reviewed and approved by the Institutional Animal Care and Use Committee (IACUC) at the China CDC animal facility and were performed in accordance with relevant guidelines and regulations. A sample of 500 μg of purified plasmid DNA of SV1.0, SV145 and SV145-10 M was suspended in 500 μl of PBS. A group of four guinea pigs were intramuscularly injected three times at Weeks 0, 2 and 4. Thereafter, guinea pigs in each group were boosted with 1 × 10^7 ^pfu recombinant Tiantan Vaccinia-vectored vaccines at six weeks after the last DNA inoculation. Sera were collected at four and six weeks after the last inoculation and stored at 4°C and -80°C for future quantification of antibodies.

### HIV-1 CN54 envelope-specific binding antibody assay

Purified HIV-1_cn54 _gp120 (more than 85% purity) was resolved in sodium bicarbonate buffer (pH 9.6) at a final concentration of 4 μg/ml, and 100 μl was added to each well of 96-well flat-bottom plates (Costar, NY). Plates were coated at 4°C overnight, then washed twice with PBS and blocked at 37°C for one hour with blocking solution (PBS containing 5% skimmed dry milk). Mice sera were serially two-fold diluted in blocking solution, and 100 μl of diluted sera was added to each well. After incubating the plates at 37°C for one hour, the plates were then washed five times with PBS-T (PBS containing 0.05% Tween20), and 100 µl/well of peroxidase-conjugated anti-mouse immunoglobulin G (diluted 1:2000 in block solution) was added and incubated for 30 minutes at 37°C. The wells were washed again, and 100 μl of OPD substrate (Cat# P9187, Sigma Aldrich) was added and incubated for approximately 10 minutes at room temperature. Color development was terminated by the addition of 50 μl/well 2 N sulfuric acid. Antibody reactivity was then determined by measuring the optical density (OD) at 492 nm with an automated plate reader (Multiscan Ascent, Thermo Corporation, Finland). Endpoint titers were determined by the last dilution whose OD was >two-fold that of the corresponding dilution of the control sera.

### Linear antibody epitope mapping

A method of PLL-ELISA was employed in this study [28,49]. PLL (poly-L-leucine, 30-70 kDa, Sigma Aldrich) was dissolved at the concentration of 40 μg/ml in the sodium bicarbonate buffer, and 50 μl/well of PLL solution was added to a 96-well ELISA plate and incubated at room temperature for one hour. The plate was washed once with PBS, incubated with 50 μl per well of 1% (v/v) glutaraldehyde (Sigma Aldrich) in PBS at room temperature for 15 minutes and washed twice with PBS. Then, 50 μl/well of each peptide of SHIVchn19 (kindly provided by the NIH Research and Reference Reagent Program, NIAIDS, NIH, Cat# 4974: The Env of SHIVchn19 is CN54 Env) in PBS was added at the concentration 10 μg/ml. The plates were incubated overnight at 4°C and then washed twice with PBS. Reactive aldehyde sites were blocked by the addition of 1 M glycine, at 200 μl/well, for one hour at room temperature. The plates were washed twice again with PBS and blocked with 1 M glycine, at 100 μl/well, for one hour at room temperature, and then they were incubated with 100 μl/well of 0.5% skimmed dry milk:0.5% gelatin (dissolved in PBS) for one hour. Mouse sera were diluted at the ratio of 1:100 in PBS containing 5% skimmed dry milk, and 50 μl/well of diluted sera was added into the plates in duplicate and incubated at 37°C for one hour. The plates were then washed four times with PBS-T, incubated with 50 μl/well of diluted HRP-linked second antibody (1:2000 diluted in PBS containing 2% skimmed dry milk, CAT# ZB-2305, Beijing Zhongshan Biotech) at 37°C for one hour, washed five times again with PBS-T and finally incubated with 50 μl/well of OPD substrate at room temperature in a dark place for about 10 minutes. The reaction was terminated with 50 μl/well 2 N sulfuric acid, and the OD value for each well was read with an automated plate reader at 492 nm. OD values of experimental settings were displayed after subtraction of the background (that is the OD value generated with sera from mock vector control groups against the corresponding peptide), and >two-fold average OD values of background were considered as positive.

### HIV virus preparation and titration

HIV-1 primary viruses were isolated from patients' peripheral blood mononuclear cells (PBMCs) by Ficoll-Paque gradient centrifugation (Amersham biosciences) and co-cultured with phytohemagglutinin- (PHA)-stimulated PBMCs from two HIV-1-seronegative human donors. The cells were maintained in RPMI 1640 medium (Gibco) containing 20 U/ml of recombinant interleukin-2 (IL-2; National Institutes of Health; Bethesda, Maryland, USA), 1% penicillin and streptomycin (P/S), 2 mM glutamine and 10% FBS. Five clade B'/C (CRF07) HIV-1 (XJDC6371, XJDC6431, XJDC0793, CBJB105, CBJB248) and four clade B' HIV-1 (020100374, 020100259, 020100311, 020100691) clinical isolates were used for this study [50]. Two clade B HIV-1 isolates (SVPB16, SVPB17), two clade C HIV-1 isolates (SVPC5, SVPC12) and three B'/C HIV-1 (CH91, CH110, CH119) tier 2 pseudotyped viruses were used in neutralizing antibody assay. There is no *env *sequence of these viruses matched mutations.

The 50% tissue culture infectious dose (TCID50) of a single thawed aliquot of each batch of virus was determined in TZM-BL cells. For TCID50 measurements, serial five-fold dilutions of viruses were made in quadruplicate wells in 96-well culture plates in a total volume of 100 μl of growth medium for a total of 11 dilution steps. Freshly trypsinized cells (10,000 cells in 100 μl of growth medium containing 75 μg/ml DEAE-dextran) were added to each well, and the plates were incubated at 37°C in a humidified environment with 5% CO_2_. After a 48-hour incubation, 100 μl of culture medium was removed from each well and 100 μl of Bright-Glo reagent (Luciferase Assay system, Promega) was added to the cells. After a two-minute incubation at room temperature to allow cell lysis, 150 μl of cell lysate was transferred to 96-well black solid plates (Corning-Costar) for measurements. The plates were read immediately with a 1420 Multilabel Counter (PerkinElmer). Wells producing relative luminescence units (RLU) >three-fold background were scored as positive. Indinavir was added to the medium at a final concentration of 1 μM to prevent progeny virion production.

### Neutralizing antibody assay

All mice and guinea pigs sera were heat-inactivated at 56°C for one hour prior to assaying. Volumes of 25 μl of sera from different immunized mice groups were diluted in 125 μl of DEAE-GM solution containing 10% heat-inactivated FBS, 50 μg/ml gentamicin and 1 μM indinavir to a 1:6 dilution. The diluted sera were further two-fold diluted as 1:12 and 1:24 in 96-well plates. Guinea pigs' sera were three-fold diluted from titer 1:10 to 1:270. Thereafter, each well received 50 μl of cell-free virus (200 TCID_50_). After one hour of incubation, 10,000 TZM-bl cells were added to each well. Plates were incubated for 48 hours. Then, 100 μl of media removed, and Bright-Glo substrate was added to each well. Percentages of RLU reduction were calculated as (1- (Average RLU of duplicates with sample sera - control wells)/(Average RLU from mock control sera - control wells)) X100%. Neutralizing antibody titers were expressed as the reciprocal of the serum dilution required to reduce RLU by 50%.

### IFN-γ ELISPOT assay

HIV-1-specific T cell responses were measured using the IFN-γ ELISPOT assay kit (BD Biosciences, United States). Plates were coated with purified anti-mouse IFN-γ at a concentration of 5 μg/ml, incubated at 4°C overnight, then washed once with RPMI1640 containing 10% FBS and 1% Penicillin-Streptomycin-L-glutamine and finally blocked for two hours at room temperature. Mouse splenocytes (2 × 10^5^) were added to wells in duplicates. Cells were stimulated with the HIV-1 CN54 ENV1 or ENV2 peptide pools at 4 μg/ml each peptide. The [SHIVchn19 (20-mer) peptides] ENV1 pool is made up of the first 43 peptides (4830-4871), and others compose the ENV2 pool (4872-4913). The positive control was stimulated with PMA at 50 ng/ml and ionomycin at 1 μg/ml, and the negative control was stimulated with medium only. The splenocytes were incubated at 37°C and 5.0% CO_2 _for 24 hours and then lysed with sterile water. Plates were washed three times with PBS-T prior to one-hour incubation with biotinylated anti-mouse IFN-γ antibody, followed by the addition of streptavidin-HRP for one hour at 37°C. Plates were washed again and developed with 100 μl of AEC substrate solution for 10~30 minutes. The reaction was stopped by washing with distilled water. IFN-γ spots were analyzed by an automated ELISPOT plate reader (ImmunoSpot, C.T.L). Spot-forming cells (SFCs) were defined as the average number of spots in duplicate wells per 10^6 ^PBMCs.
